# Cinematic Rendering in Mixed-Reality Holograms: A New 3D Preoperative Planning Tool in Pediatric Heart Surgery

**DOI:** 10.3389/fcvm.2021.633611

**Published:** 2021-02-09

**Authors:** Pia Gehrsitz, Oliver Rompel, Martin Schöber, Robert Cesnjevar, Ariawan Purbojo, Michael Uder, Sven Dittrich, Muhannad Alkassar

**Affiliations:** ^1^Department of Pediatric Cardiology, University Hospital Erlangen, Friedrich-Alexander University Erlangen-Nürnberg (FAU), Erlangen, Germany; ^2^Institute of Radiology, University Hospital Erlangen, Friedrich-Alexander University Erlangen-Nürnberg (FAU), Erlangen, Germany; ^3^Department of Pediatric Cardiac Surgery, University Hospital Erlangen, Friedrich-Alexander University Erlangen-Nürnberg (FAU), Erlangen, Germany

**Keywords:** mixed-reality, cinematic rendering, 3D printing, preoperative planning, pediatric heart surgery, congenital heart disease

## Abstract

Cinematic rendering (CR) is based on a new algorithm that creates a photo-realistic three-dimensional (3D) picture from cross-sectional images. Previous studies have shown its positive impact on preoperative planning. To date, CR presentation has only been possible on 2D screens which limited natural 3D perception. To depict CR-hearts spatially, we used mixed-reality technology and mapped corresponding hearts as holograms in 3D space. Our aim was to assess the benefits of CR-holograms in the preoperative planning of cardiac surgery. Including 3D prints allowed a direct comparison of two spatially resolved display methods. Twenty-six patients were recruited between February and September 2019. CT or MRI was used to visualize the patient's heart preoperatively. The surgeon was shown the anatomy in cross-sections on a 2D screen, followed by spatial representations as a 3D print and as a high-resolution hologram. The holographic representation was carried out using mixed-reality glasses (HoloLens®). To create the 3D prints, corresponding structures were segmented to create STL files which were printed out of resin. In 22 questions, divided in 5 categories (3D-imaging effect, representation of pathology, structure resolution, cost/benefit ratio, influence on surgery), the surgeons compared each spatial representation with the 2D method, using a five-level Likert scale. The surgical preparation time was assessed by comparing retrospectively matched patient pairs, using a paired *t*-test. CR-holograms surpassed 2D-monitor imaging in all categories. CR-holograms were superior to 3D prints in all categories (mean Likert scale 4.4 ± 1.0 vs. 3.7 ± 1.3, *P* < 0.05). Compared to 3D prints it especially improved the depth perception (4.7 ± 0.7 vs. 3.7 ± 1.2) and the representation of the pathology (4.4 ± 0.9 vs. 3.6 ± 1.2). 3D imaging reduced the intraoperative preparation time (*n* = 24, 59 ± 23 min vs. 73 ± 43 min, *P* < 0.05). In conclusion, the combination of an extremely photo-realistic presentation via cinematic rendering and the spatial presentation in 3D space via mixed-reality technology allows a previously unattained level of comprehension of anatomy and pathology in preoperative planning.

## Introduction

Due to the complex and highly individual anatomy of patients with congenital heart disease (CHD), it is essential to have precise preoperative planning and good morphologic imaging for surgical success. Currently, three-dimensional (3D) imaging offers the most realistic representation of cardiac structures, and has therefore gained importance in recent years (1–5).

3D images are generated from two-dimensional (2D) cross-sectional images produced using computed tomography (CT) and magnetic resonance imaging (MRI). There are two methods for generating 3D images from 2D datasets: (1) creating 3D segmentation by manually selecting interesting structures, and (2) calculating a 3D image automatically, based on rendering algorithms. Siemens Healthineers has developed a new volume rendering technique called cinematic rendering (CR). CR generates a more photo-realistic 3D depiction than previously used rendering algorithms, by imitating natural light interactions (3, 6). Multiple previous studies have confirmed that CR provides a more photo-realistic view and improvements in shape and depth perception compared with cross-sectional imaging or volume rendering (7–10).

Until recently, 3D-rendered images can be presented only on a 2D screen. Currently, it is possible to present these 3D images in 3D space with either physical or virtual 3D imaging. Physical 3D imaging is generated by producing a 3D-printed model from a manually-generated 3D image. Virtual 3D imaging is generated by creating a hologram by using mixed-reality technology. The latest development is an application that integrates CR and mixed-reality techniques for use with the HoloLens® (Microsoft, Redmond, USA) (11). The current gold standard for spatial imaging in preoperative planning is 3D printing. However, it has mostly been described in case reports and systematic reviews of its advantages are still rare. Furthermore, a significant benefit compared to 2D imaging regarding the overall surgery time could not be shown yet for 3D printing. Therefore, we compared both 3D imaging methods additional to the standard preoperative imaging on 2D screen.

The aim of this study was to determine the benefits of spatial representation of CR-reconstructed heart structures in the preoperative planning of pediatric heart surgery. By including 3D prints, a direct comparison of two spatially resolving display methods was possible.

## Materials and Methods

This prospective study was performed in accordance with the Guidelines for Good Clinical Practice. All CT and MRI datasets were accessed with permission through informed consent from both parents; in no case were the images taken exclusively for this study.

The patients were recruited between February and September 2019. All patients who underwent cross-sectional imaging in preparation for surgery were included in the study. The decision to perform cross-sectional imaging was based on comprehensive echocardiography performed previously. Because high-quality cross-sectional images from MRI and CT are equally suitable for 3D imaging (2–4, 6, 7, 12–15), cross-sectional images were recorded using MRI or CT depending on the clinical question. If morphology was the only question, CT was used. If there were additional functional questions, MRI was carried out.

### Data Acquisition

The CT scans were performed during the post venous phase, after injecting contrast medium peripheral. The images were acquired in 0.6 mm slices, using either a second-generation 128-slice dual-source CT scanner (SOMATOM Definition Flash; Siemens Healthcare GmbH, Erlangen, Germany) or a third-generation 192-slice dual-source CT scanner (SOMATOM Definition Force; Siemens Healthcare, Erlangen, Germany). Modern low-dose (0.2–0.5 mSv) protocols were used. The MRI datasets were collected in diastolic heart phase, in a whole-heart sequence, in 0.8 mm slices, with a 1.5-Tesla MRI-scanner (MAGNETOM Aera; Siemens Healthcare GmbH, Erlangen, Germany).

### Spatial View

To set up the spatial representation, datasets were exported from the advanced visualization imaging software, *syngo*.via (Version VB30A; Siemens Healthcare GmbH, Erlangen, Germany), and saved in the standard “Digital Imaging and Communications in Medicine” (DICOM)-format.

**Figure 1:**
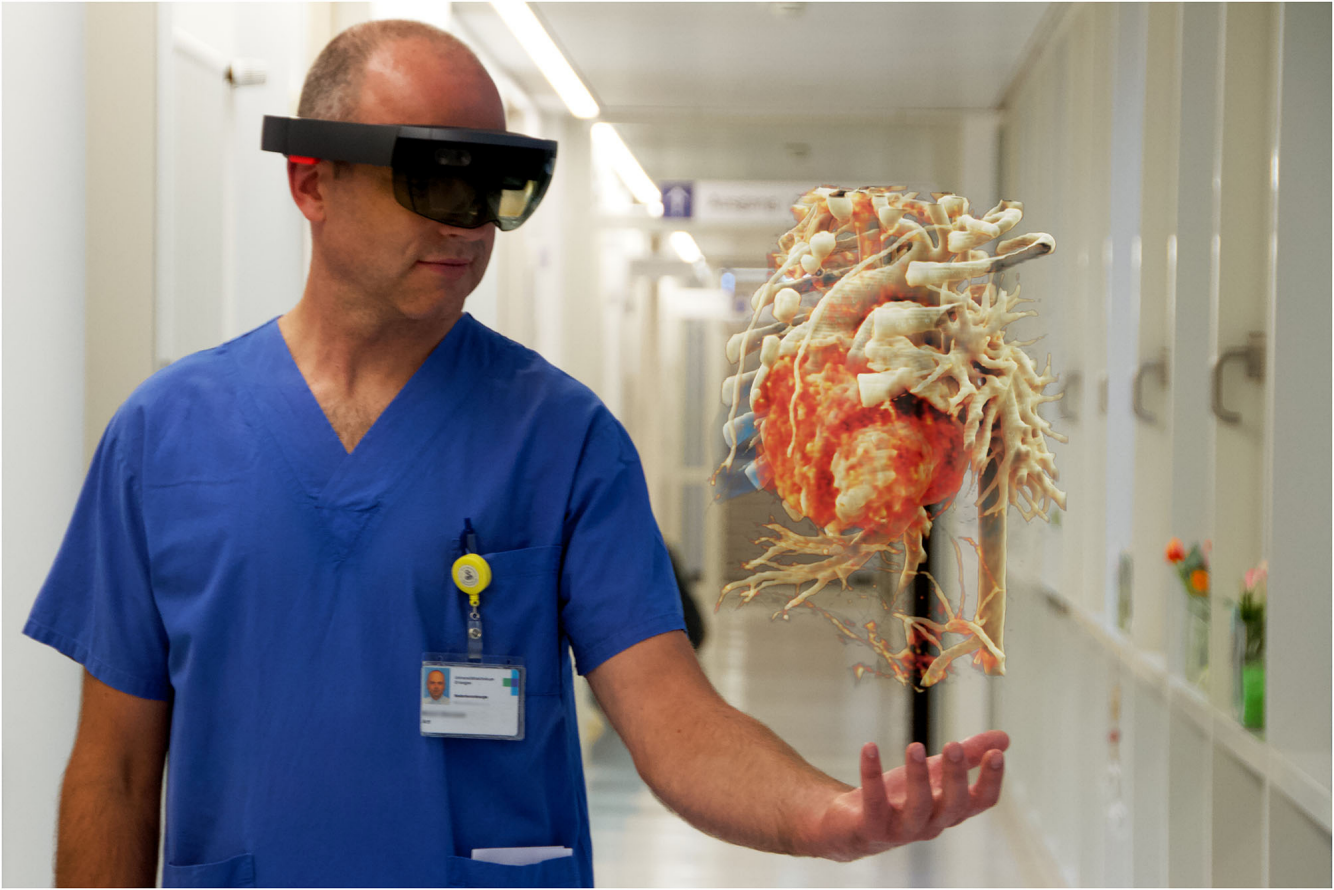
Surgeon looking at a CR-hologram of a patient's heart with dextro-transposition of the great arteries using HoloLens®. The figure illustrates an image for which the view of the surgeon through the HoloLens® was combined with a photo in which he is working with the HoloLens®.

DICOM-data were used to visualize the heart directly in the cinematically rendered view with the newly developed prototype mixed-reality *syngo*.via application “Cinematic Reality.” The new *syngo*.via application generated a cinematically rendered hologram (CR-hologram), which could be viewed with the HoloLens®. The hologram was projected in a firmly fixed position in the room, so the observer could walk around the heart and examine it from every side. Figure 1 shows a surgeon looking at a CR-hologram through the HoloLens®.

To create a realistic 3D-printed model from cross-sectional images, various pre-processing steps were necessary. First, DICOM-data were exported into the open-source software, 3D Slicer (Version 4.11; http://www.slicer.org). In this software, the image was segmented based on an adjustable threshold chosen so that only the voxels of interest were marked. The marking depended on the master volume intensity range of the individual voxels. In addition to the whole heart, neighboring vessels were marked that were relevant to later surgery.

**Figure 2:**
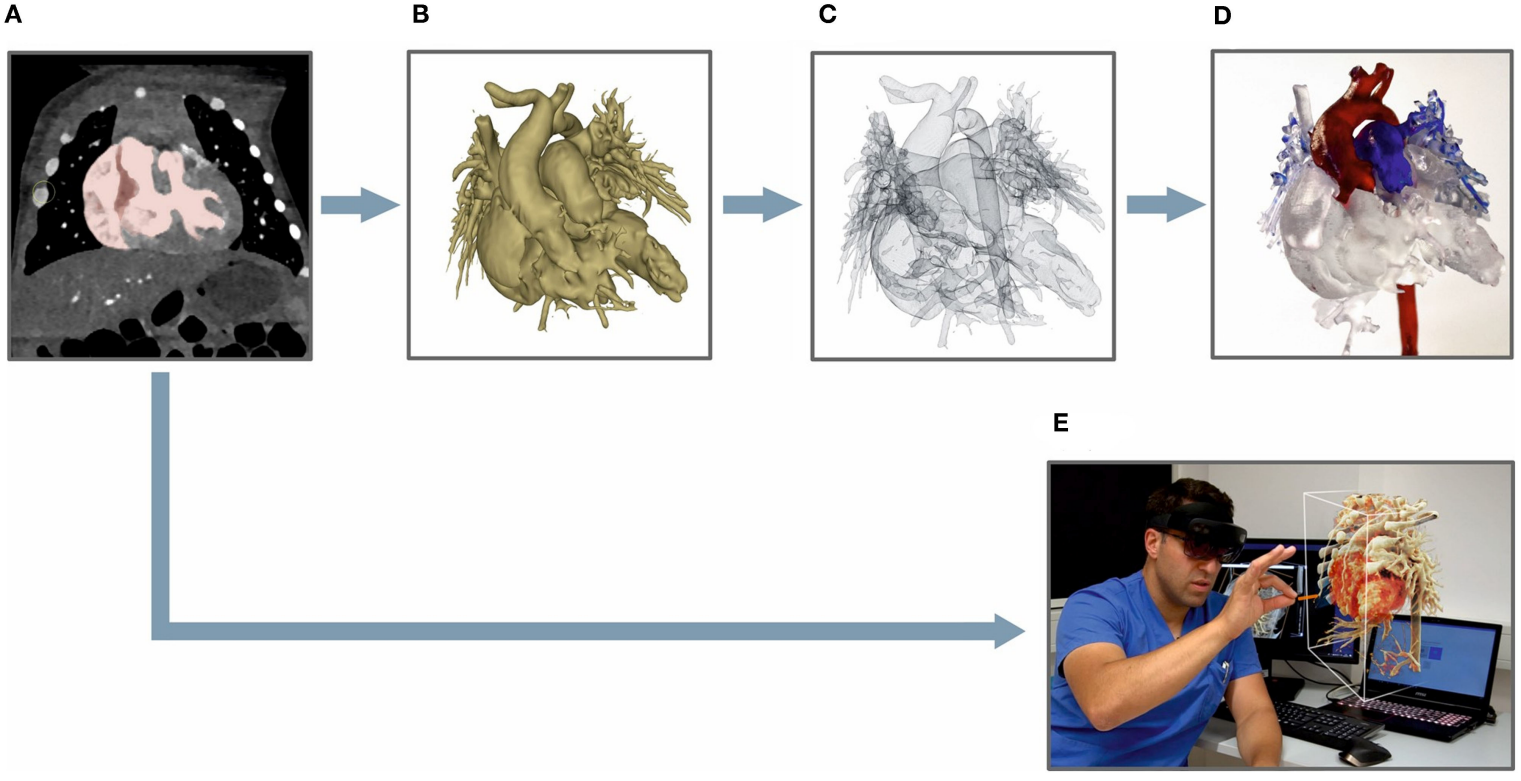
Processing steps for generating a 3D-printed model **(A–D)** vs. a CR-hologram **(E)**: **(A)** DICOM-data viewed in the 3D Slicer application, marked based on an appropriate threshold; **(B)** a segmented 3D model; **(C)** a 3D model as an STL file; **(D)** a finished printed 3D model; **(E)** cross-sectional images converted to a CR-hologram.

The created model was saved in standard tessellation language (STL) file format, which was compatible with the 3D printer. Next, the model was produced by 3D printer (Form 2, Formlabs, Sommerville, USA) out of resin at a resolution of 0.1 mm layers using standardized printing plans. As a post-processing step, important structures such as coronary arteries were marked in different colors to facilitate orientation. An overview of the main steps for generating a 3D-printed model in comparison to a CR-hologram is given in Figure 2. The imaging processing time for each technique was recorded.

### Study Design

First, the patient's cross-sectional images were presented on a 2D screen, which was previously the common technique for presenting preoperative imaging material. The CR-hologram, and subsequently the 3D-printed model, were then presented to the surgeon in an upright position. To provide a comparison of the newly introduced spatial 3D-imaging techniques and the representation on the 2D screen, the surgeons were asked to complete a questionnaire for each spatial 3D-imaging technique. The two 3D techniques were also directly compared with each other.

### Questionnaire

In total, the questionnaire comprised 22 items, each rated on a 5-point Likert scale. The five response options ranged from “clearly superior” (5 points) to “clearly inferior” (1 point). The questionnaire items were structured in five subgroups to provide a better overview: 3D-imaging effect, representation of the pathology, anatomical structure resolution, cost/benefit ratio, and influence on the surgery (**Table 2**). The reliability of the different items of each dimension was checked using Cronbach's alpha.

### Analysis of Surgical Preparation Time

In order to investigate the influence of preoperative spatial representation on the surgical procedure, we compared intraoperative preparation times. Intraoperative preparation time was defined as the time from the initial cut until the first vessel was clamped. The intraoperative preparation times for study participants were compared with intraoperative preparation times for patients with matching characteristics for whom preoperative imaging in 3D space was not used. The patients were matched for age, weight, operative procedure, previous operations, and the general state of health preoperatively (Table 1). As intraoperative preparation time was analyzed, only patients who underwent the same surgical procedures with the same complexity were matched. Additionally, the intraoperative preparation times of the matched patient pairs were analyzed after being divided into groups in which the “facilitation of preparation” was, respectively, rated of excellent benefit (5 points on the Likert scale) or not (≤ 4 points). A Wilcoxon test was performed to compare the two groups because the *F*-test showed no equality of variance for the “advantageous” group.

**Table 1A:** Demographic information about the analyzed patient population.

**Patient characteristics**		**Cases**	**Matched controls**
Number		*n* = 26	*n* = 24
Gender	Male	61.5%	58.3%
	Female	38.5%	41.7%
Age (years)		2.0 ± 3.9	2.0 ± 4.7
Weight (kg)		10.5 ± 12.9	10.7 ± 15
Heart lung machine	Yes	92.3%	95.8%
	No	7.7%	4.2%
Access	Median sternotomy	84.6%	87.5%
	Posterolateral	15.4%	12.5%
Previous operations	Yes	34.6%	29.2%
	No	65.4%	70.8%

### Statistical Evaluation

Statistical analyses were performed using IBM SPSS statistics (Version 21; IBM, Armonk, USA). Results of the questionnaires were expressed as mean values with standard deviation (SD). Each questionnaire item was analyzed separately and summarized in the five subgroups.

A paired *t*-test was performed to compare the questionnaire results (Likert scale data) of CR-holograms with those of 3D-printed models. As Jeffrey and Norman have shown, parametric tests are superior to non-parametric tests when analyzing Likert scale data (16, 17). To compare the intraoperative preparation times between the patients and matched controls a paired *t*-test was used. The statistical significance level was defined as *P* < 0.05 for all analyses.

The contrast-to-noise ratio (CNR) and signal-to-noise ratio (SNR) were determined independently for CT and MRI via regions of interest (ROIs) using the software *syngo*.via. The SNR was calculated by dividing the mean signal intensity of the aorta in cross-section (*ROI* = 1.2 ± 0.1 mm^2^) by the SD of the extra thoracic background noise (*ROI* = 2.2 ± 0.1 cm^2^) measured from the air surrounding the patient. For the CNR the mean signal intensity of the left ventricular muscle (*ROI* = 1.2 ± 0.1 mm^2^) was subtracted from the mean signal intensity of the aorta and afterwards divided by the SD of the background noise.

## Results

We recruited 26 patients with an average age of 2.0 ± 3.9 years. Of the surgeries, 77% concerned mainly outer cardiovascular structures (e.g., great vessels) and 23% inner cardiovascular structures (e.g., valves). Twenty-four patients underwent CT as preoperative imaging modality and 2 patients MRI. CT and MRI datasets were of comparable quality (CNR: MRI: 16.4 ± 1.4, CT: 13.7 ± 6.4; SNR: MRI: 19.8 ± 2.1, CT: 20.28 ± 8.5). Cross-sectional images for all 26 patients were of high quality without artifacts and could be used successfully for both rendering and 3D printing. Further demographic information about the patients and the conducted surgeries is shown in Table 1. The cross-sectional imaging was taken on average 13 days before the surgery took place. The patients' images were of comparable quality; in particular, no difference between the cases and the matched control patients could be identified (CNR: cases: 14.0 ± 6.4, control: 14.5 ± 7.8; SNR: cases: 20.3 ± 8.5, control: 21.9 ± 12.3).

**Table 1B:** Demographic information about the analyzed patient population.

**Case**	**Imaging**	**Cases**			**Matched controls**		
		**Main diagnosis and kind of operation**	**Age (years)**	**Weight (kg)**	**Main diagnosis and kind of operation**	**Age (years)**	**Weight (kg)**
1	CT	Single ventricle (1)	<1	3.3	Single ventricle (1)	<1	2.7
2	CT	Pulmonary atresia + VSD^a^ (1)	<1	8.0	Pulmonary atresia + VSD (1)	2	12.0
3	CT	CoA^b^ (2)	<1	3.1	CoA (2)	<1	4.7
4	CT	Ductus arteriosus aneurysm (3)^*^	<1	3.4	Ductus arteriosus aneurysm (3)	<1	5.6
5	CT	Single ventricle (4)	<1	3.5	Single ventricle (4)	<1	3.5
6	CT	Pulmonary atresia + VSD (1)	4	18.4	Pulmonary atresia + VSD (1)	2	14.7
7	CT	Single ventricle (1)	<1	3.0	ToF^c^ (1)	<1	3.0
8	CT	Truncus arteriosus (1)	<1	3.8	Truncus arteriosus (1)	<1	3.1
9	CT	Single ventricle (5)	<1	7.7	Single ventricle (5)	<1	6.4
10	CT	d-TGA^d^ (6)^*^	<1	3.5	d-TGA (6)	<1	3.3
11	CT	AVSD^e^ (7)^*^	2	11.0	AVSD (7)	1	8.3
12	CT	Aortopulmonary window (7)^*^	<1	4.1	Aortopulmonary window (7)	<1	3.4
13	MRI	Pulmonary valve disease (8)^*^	13	52.0	Pulmonary valve disease (8)	14	48.0
14	CT	Tracheomalacia (9)	2	7.7	Tracheomalacia (9)	<1	7.0
15	CT	CoA (2)^*^	<1	4.6	CoA (2)	<1	3.7
16	CT	ToF (7)^*^	<1	6.8	AVSD (7)	<1	7.0
17	CT	CoA (2)^*^	3	15.0	CoA (2)	5	20.0
18	MRI	Hypoplasia of the aortic root (8)	13	50.0	Aortic valve disease (8)	19	65.0
19	CT	ALCAPA^f^ (10)^*^	<1	5.2	ALCAPA (10)	<1	3.7
20	CT	Single ventricle (11)	<1	6.9	Hypoplasia of the aorta (11)	<1	4.5
21	CT	Single ventricle (12)	5	13.5	Single ventricle (12)	5	15.0
22	CT	Tracheomalacia (13)	<1	5.8	–	–	–
23	CT	cc-TGA^d^ + multiple comorbidities (14)	10	22.6	–	–	–
24	CT	Single ventricle (4)	<1	2.8	Single ventricle (4)	<1	3.5
25	CT	ToF (1)	<1	4.3	Pulmonary atresia + VSD (1)	<1	6.2
26	CT	d-TGA (6)	<1	4.2	d-TGA (6)	<1	3.0

a Ventricular septal defect;

b Coarctation of the aorta;

c Tetralogy of Fallot;

d dextro/congenital corrected-transposition of the great arteries;

e Atrioventricular septal defect;

f Anomalous left coronary artery from the pulmonary artery.

*In brackets: kind of operation: (1) Shunt between right ventricle and pulmonary artery; (2) end-to-end anastomosis; (3) aneurysm resection; (4) aortopulmonary shunt; (5) upper cavopulmonary connection; (6) atrial switch; (7) closure with bovine pericardial patch; (8) valve replacement; (9) trachea reconstruction; (10) coronary artery transfer; (11) aortic arch reconstruction; (12) DORV-correction; (13) aortopexy; (14) VSD closure*.

* *Patients rated with no benefit of holographic presentation for the surgeon's preparation*.

The average time required to create a CR-hologram was 9.0 ± 2.1 min. The generation of a 3D-printed model required an average of 141.8 ± 27.7 min for the pre-processing steps, 240–1,185 min for the printing process (depending on size and complexity), and an average of 38.2 ± 10.0 min for the post-processing steps.

### The HoloLens® Surpassed 2D Imaging in All Subgroups

The items of the questionnaire were divided into five subgroups. The group affiliation was tested using Cronbach's alpha, which showed good inter-item correlation without unnecessary redundancy for all subgroups: 3D-imaging effect (CR-holograms: 0.72; 3D print: 0.81), representation of the pathology (CR-holograms: 0.87; 3D print: 0.88), anatomical structure resolution (CR-holograms: 0.81; 3D print: 0.80), cost/benefit ratio (CR-holograms: 0.70; 3D print: 0.73), influence on surgery (CR-holograms: 0.88; 3D print: 0.88) (18).

The analysis of the questionnaire results showed benefits for CR-holograms compared with 2D imaging in all five subgroups (Table 2). Further examination of the single items showed no benefit only for two items: assessment of intracardial structures (3.3 ± 1.1) and the coronaries (3.4 ± 1.4).

**Table 2:** Evaluation of CR-holograms and 3D-printed models divided in subgroups.

**Items**	**CR-holograms vs. 2D imaging**	**3D printing vs. 2D imaging**	
	**mean (SD)**	**mean (SD)**	***P*-value**^**#**^
**3D-imaging effect**	**4.4 (0.8)**	3.4 (1.1)^*^	**0.000**^**§**^
Comparison with CR on 2D screen	4.1 (1.3)	2.7 (1.5)^*^	0.000^§^
Sufficient visualization options	4.5 (1.0)	3.7 (1.4)	0.001^§^
Sufficient quality	4.4 (1.1)	3.5 (1.4)	0.000^§^
Improved depth perception	4.7 (0.7)	3.7 (1.2)	0.000^§^
**Representation of the pathology**	**4.4 (0.9)**	**3.6 (1.2)**	**0.001**^**§**^
All necessary areas presented	4.3 (1.1)	3.5 (1.4)	0.001^§^
Important details not hidden	3.9 (1.5)	3.2 (1.6)^*^	0.010^‡^
Improved comprehensibility	4.6 (0.9)	3.9 (1.3)	0.008^§^
Adequate pathology assessment	4.7 (0.7)	4.0 (1.2)	0.003^§^
**Anatomical structure resolution**	**4.3 (0.5)**	**3.8 (0.7)**	**0.000**^**§**^
Confluence of vessels	4.8 (0.5)	4.4 (0.9)	0.022^‡^
Out-flowing vessels	4.7 (0.6)	4.2 (1.0)	0.005^§^
Aortic arch	4.9 (0.3)	4.9 (0.3)	1.000^†^
Coronaries	3.4 (1.4)^*^	2.5 (1.3)^*^	0.003^§^
Pulmonary veins	4.7 (0.5)	4.1 (1.2)	0.023^‡^
Atrial appendages	4.8 (0.4)	4.4 (0.7)	0.010^‡^
Structures of the inner heart	3.3 (1.1)^*^	3.0 (1.3)^*^	0.397^†^
Neighboring structures	4.3 (1.0)	3.1 (1.4)^*^	0.000^§^
**Cost/benefit ratio**	**4.5 (0.7)**	**4.0 (0.9)**	**0.012**^‡^
Appropriate expenditure of time	4.5 (1.0)	3.9 (1.1)	0.03^‡^
Educational potential	4.7 (0.6)	4.5 (0.8)	0.056^†^
Adequate costs and benefits	4.2 (1.1)	3.7 (1.3)	0.020^‡^
**Influence on the surgery**	**4.4 (0.8)**	**3.8 (1.0)**	**0.004**^**§**^
Concordant intraoperative view	4.7 (0.7)	4.1 (1.0)	0.006^§^
Facilitation of preparation	4.5 (0.8)	3.9 (1.0)	0.003^§^
Positive impact on surgery time	4.2 (1.0)	3.5 (1.2)	0.016^‡^

# *paired t-test for analysis*,

† *No significant difference*,

‡ *p < 0.05*,

§ p < 0.01.

* *presentation on 2D screen superior to presentation in 3D space. The bold values correspond to the five subgroups that were evaluated. The corresponding items from which a subgroup was composed are listed below the respective bold subgroup*.

### Neighboring Structures and Holographic Visibility Are Essential for the Superiority of the HoloLens®

**Figure 3:**
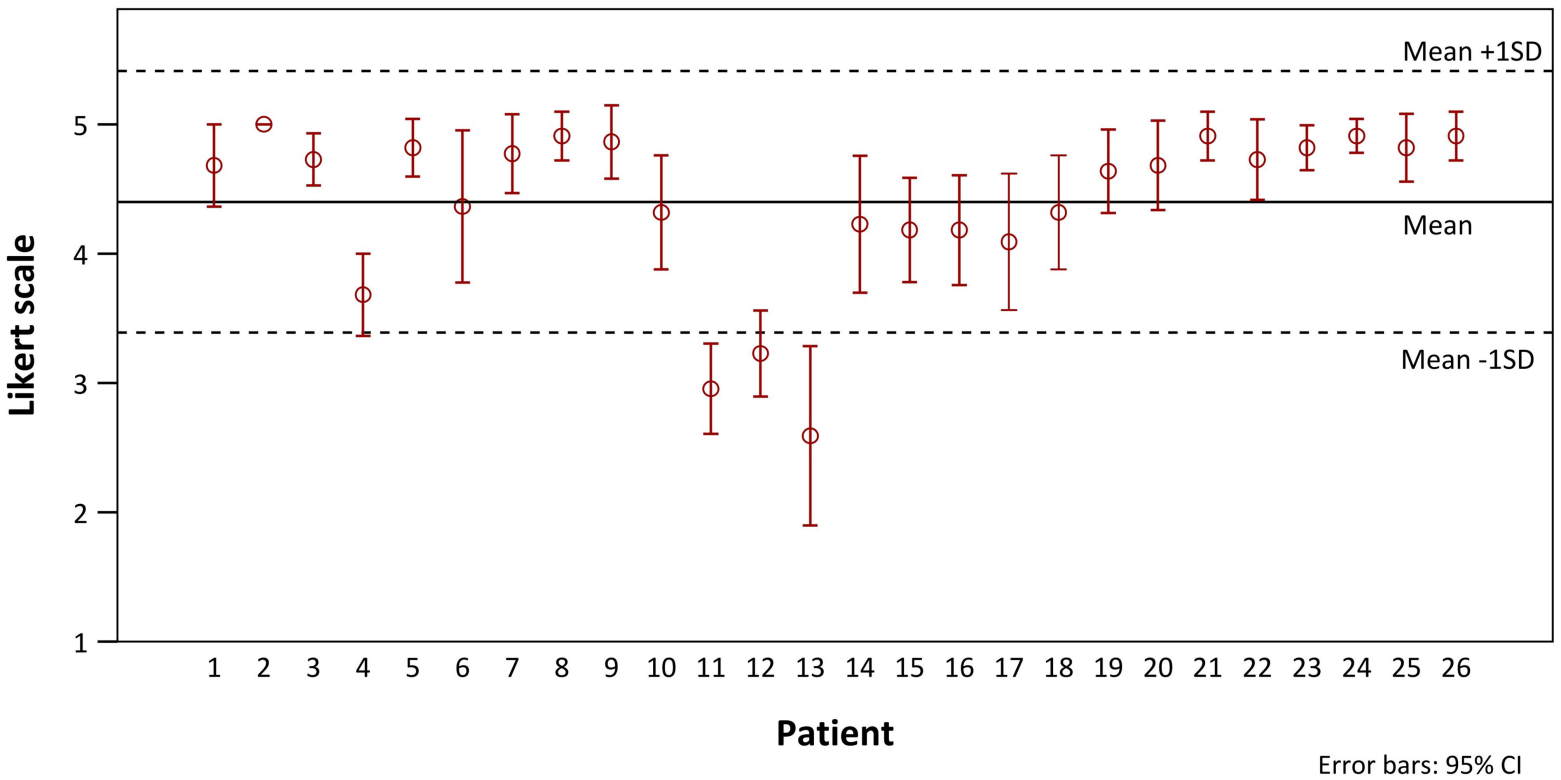
Distribution (means with 95% confidence intervals) of the Likert scale ratings of CR-holograms over the progress of the study.

**Figure 4:**
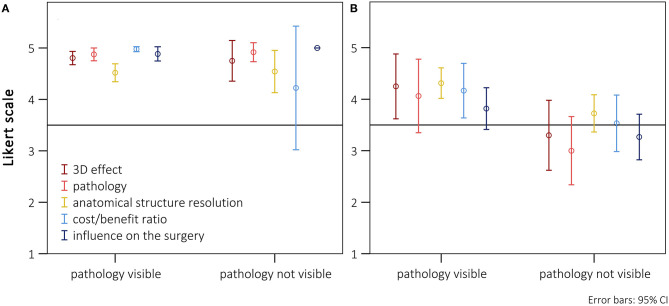
Presentation of the scores (means with 95% confidence intervals) separately listed for the five subgroups, divided between surgeries where the main pathology was visible or not using the HoloLens®. **(A)** Cases with an excellent rating for preparation-time benefit (*Likert scale* = 5; *n* = 17). **(B)** Cases without an excellent rating for preparation-time benefit (Likert scale <5; *n* = 9).

We investigated potential differences between different patient cohorts. The Likert scale values of all patients in chronological order are shown in Figure 3. There was no indication of a chronological dependency of the ratings. In 3 of 26 patients, no benefit for the average values could be identified in comparison with 2D imaging (patients 11, 12, and 13). On closer examination it became apparent that these patients underwent a repair of inner cardiovascular structures that cannot be adequately presented via CR-holograms. Additionally, for these cases the usefulness of the hologram for the preparation received no excellent ratings (3.6 ± 0.5). To assess whether these two aspects (neighboring structures and holographic visibility) influenced the overall evaluation, the patients were evaluated after separation into groups that, respectively, did and did not benefit from the representation of the pathology. There was only a benefit for the representation of intracardial pathologies when additional vascular surgery aspects were present. In Figure 4A, only patients with an excellent rating of the usefulness for preparation were included (*Likert scale* = 5, *n* = 17). No disadvantage in the overall rating was seen, even if the main pathology could not be presented holographically. For patients for whom the preparation was rated worse, a significant deterioration of the ratings in all subgroups could be shown (Figure 4B, *n* = 9). A particularly poor evaluation could be shown for patients in whom, additionally, the holographic presentation of the pathology was not possible.

### Superiority of the HoloLens® Over Previously Used 3D-Imaging Techniques

The CR-holograms were rated significantly higher than the 3D-printed models in all categories (Table 2). Nevertheless, clear differences in the ratings of the individual subgroups could be identified. Concerning cost/benefit ratio, only a small benefit could be shown for the holographic presentation compared to 3D-printed models (CR-holograms: 4.5 ± 0.7; 3D print: 4.0 ± 0.9; *P* < 0.05), while no significant difference between the 3D-imaging methods was observed for use in education. The surgeons rated the time expenditure for 3D printing only a bit higher. In the overall evaluation of the anatomical structure resolution, CR-holograms showed significantly better results (CR-holograms: 4.3 ± 0.5; 3D print: 3.8 0.7; *P* < 0.05). Nevertheless, both evaluated 3D-imaging methods showed no benefit compared with 2D imaging regarding the representation of the coronaries as well as the intracardial structures. However, CR-holograms were rated significantly higher than 3D-printed models for the representation of the coronaries. Concerning the remaining anatomical structures, only a small significant difference could be observed between the two spatial representation methods, especially considering large vessel structures. While the benefit of CR-holograms in representing the pathology was clearly higher compared with 2D imaging, for 3D printing the benefit was only marginal (CR-holograms: 4.4 ± 0.9; 3D print: 3.6 ± 1.2; *P* < 0.05). In the assessment of the 3D-imaging effect, the HoloLens® was clearly superior to 3D printing (CR-holograms: 4.4 ± 0.8; 3D print: 3.4 ± 1.1; *P* < 0.05).

### Significant Shortening of the Intraoperative Preparation Time

We evaluated the measurable influence of spatial 3D methods in preoperative planning on the course of surgery by analyzing the intraoperative preparation times. Two cases could not be included, because no suitable case could be found for comparison, due to the complexity of the respective surgery. In five cases, the main diagnosis of cases and matched controls differed, but the operative procedure and other conditions were the same in these patients.

**Figure 5:**
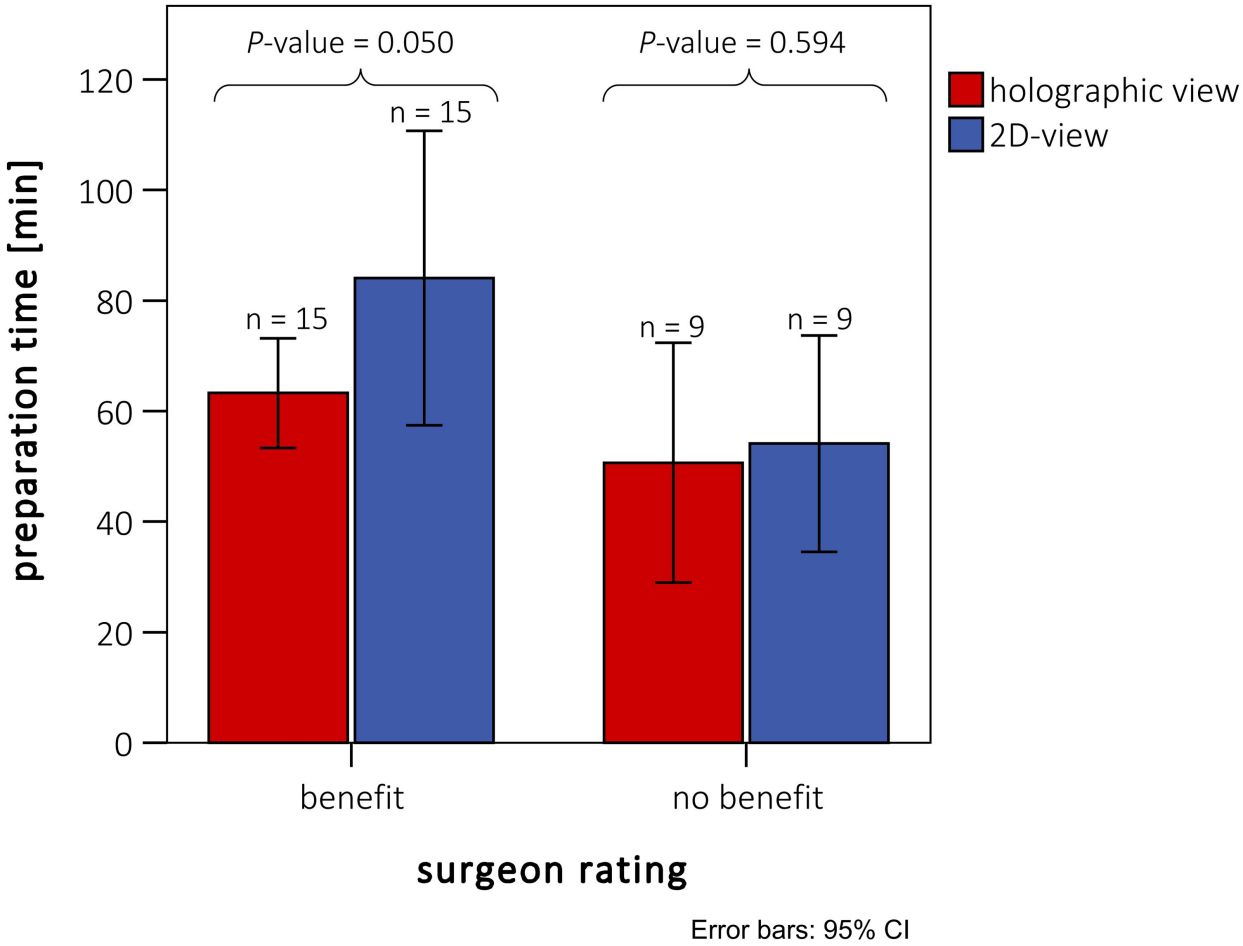
Intraoperative preparation times of patients with preoperative holographic representation (red) and matched patient pairs (blue) without spatial representation. Ratings are divided between cases rated with and without benefit for preparation by the surgeons. Patients rated with “no benefit” did not differ from the rest either in diagnosis or in kind of surgery (see also Table 1B).

The mean intraoperative preparation time was 58.5 ± 22.6 min (*n* = 24, minimum: 23 min, maximum: 104 min), when spatial 3D models were used for preoperative planning. In contrast, the preoperative planning for the relevant control group was carried out completely on a 2D monitor, and no representation in 3D space was used. The control intraoperative preparation time was 72.8 ± 43.1 min (*n* = 24, minimum: 24 min, maximum: 186 min). The intraoperative preparation times of the patients who received preoperative planning based on 3D-printed models and CR-holograms were significantly lower compared with the control group (*P* < 0.05). To examine whether this significant shortening corresponded with the surgeon's assessment, we performed an additional analysis. The matched patient pairs were separated in two groups according to the surgeon's estimate (benefit/no benefit). The preparation time was then compared between the patients and controls in these smaller groups. As illustrated in Figure 5, this conformity was good. In the 15 patients rated “advantageous” by the surgeon, the preparation time for the study patients was just significant shorter than the measured preparation time for the controls. In the nine patients rated “non-advantageous” by the surgeon, there was no significant benefit measured for the preparation time.

## Discussion

Recent developments made the use of CR in 3D space possible for the first time. This study is the first to compare CR-holograms with previously used imaging techniques in which the images were projected onto a 2D screen (CT or MRI). Precise morphological imaging is essential for surgical success, especially in complex cases.

**Figure 6:**
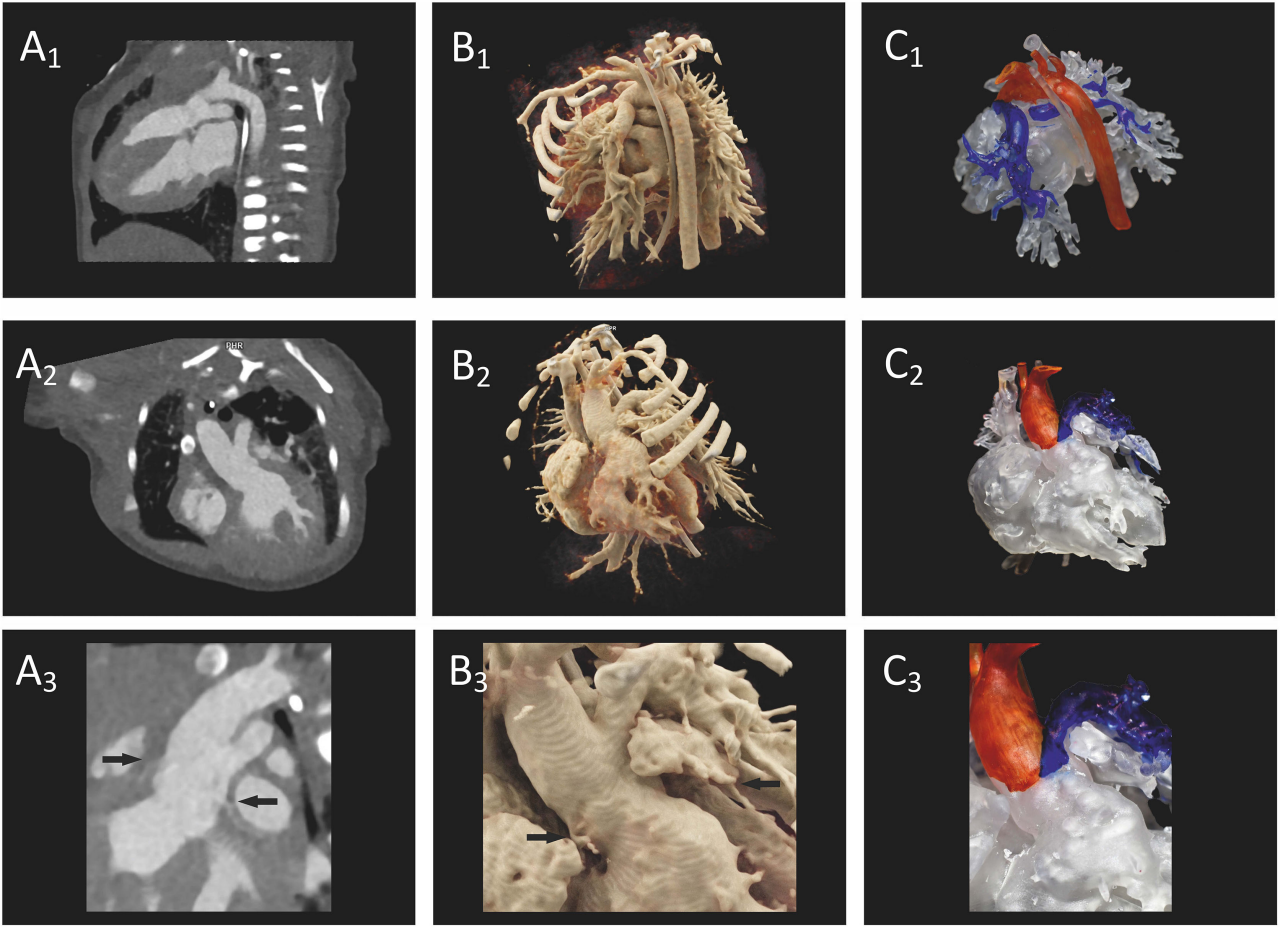
Preoperative imaging of a patient's heart with truncus arteriosus communis from two different perspectives. **(A1–A3)** A direct comparison of CT on a 2D screen (2D imaging), **(B1–B3)** cinematically rendered hologram, and **(C1–C3)** 3D printing. In **(A3)**, the coronaries (arrows) show only a weak contrast, but the 3D view can be demonstrated as a cinematically rendered hologram **(B3)**. A reconstruction of the coronary structure for 3D printing was not possible in this case **(C3)**.

A direct comparison of CR-holograms to 2D imaging and 3D printing quickly reveals the superiority of the photo-realistic 3D holographic representation (Figure 6). The holographic representation by the HoloLens® surpassed the standard representation on a 2D screen in all five analyzed parameters (Table 2). Furthermore, the assessment of an already established method of 3D imaging, 3D printing, was used as a spatial comparison method (2, 12–15, 19–21). The questions used to create the questionnaire were carefully selected. The absence of unnecessary redundancy and good inter-item correlation in the subgroups were confirmed using Cronbach's alpha, validating the selection criteria. MRI was performed in two cases because additional functional parameters were needed. The quality of these images did not differ from that of CT. Furthermore, the possibility that discrepancies in the results arose from differences between the cases and the controls in the quality of the underlying images could be rejected.

### CR-Holograms as the Most Realistic Representation Method

In fields like engineering or design review, the fundamental advantage of 3D imaging in 3D space is already proven. Spatial 3D representation has been established for many years in those areas of expertise. It has already been shown that the spatial 3D presentation improves shape and depth perception, reduces mental workload, and makes it possible to complete tasks faster and with higher quality results (22, 23). In accordance with these observations, our results showed the advantage of using spatial 3D imaging as, the most significant improvement in preoperative planning by enhancing depth perception and the representation of the pathology (Table 2). These aspects were rated better for CR-holograms than for 3D-printed models, even though the 3D prints correspond in dimensions and anatomical presentation exactly to the patient's heart and have a haptic advantage. The main difference between the 3D print and a real heart is the representation of surface features like color, texture, and lighting characteristics. The absence of these realistic features makes additional mental transformational work necessary since an intuitive recognition intraoperatively is not possible. In contrast, CR makes it possible to create a 3D image which strongly corresponds to the familiar intraoperative tissue texture. This is achieved by using an algorithm that takes the interaction between light photons and human tissue into account (3, 6–10). This could already prove to be a significant advantage, especially in shape and depth perception, over previously used rendering algorithms (e.g., volume rendering) when presented on 2D screen (7–10). However, our results showed that CR on a 2D screen improved the 3D perception so that it could show results even equal to those obtained using 3D printing (Table 2). This explains the consistently excellent review of the 3D perception of the holographic representation of CR-images. The combination of the realistic spatial 3D view with an extremely realistic rendering algorithm clearly improved depth perception and provided a better delineation of complex anatomical structures.

It is known that repeatedly visually presented objects can be processed much faster than unknown structures (24). The exact preoperative visualization of the operative field allows the surgeon to plan the operation's steps directly. Therefore, the aim of preoperative imaging is to provide the surgeon a virtual operative field in advance that is as close to reality as possible. Our study showed that the intraoperative findings corresponded to the preoperative images significantly better when the latter were presented as CR-holograms than when visualized on a 2D screen or by 3D printing (Table 2). We assume that this is the reason for the facilitated visual comprehension of the pathology.

The only barriers to easier comprehension of pathologies using CR-holograms were structures which were either not imaged in the primary dataset or could not be presented holographically (intracardial structures). The lack of information in the primary data set is most evident in the representation of the coronary arteries. The representation is dependent on the perfusion and the phase in which the single shot was taken. Since the images were not taken especially to display the coronary arteries, contrast was lacking in some patients and this limited the coronary arteries' visibility. Nevertheless, CR-holograms exhibited an advantage over 3D printing in cases with limited coronary representation in raw 2D images. In these cases, 3D printing is inferior to 2D representation, because a weak coronary contrast makes 3D reconstruction impossible. On the other hand, CR-holograms enable weakened coronary visibility, which allows equivalent visibility of coronaries as in 2D images (Figure 6). Apart from this, it is currently not possible to view intracardial structures using the HoloLens®. However, even in cases in which intracardial pathology is the main diagnosis, surgical preparation could be facilitated by the presentation of outer cardiovascular and neighboring structures.

Comparing the distribution of all ratings for the individual patients, in three cases the holographic presentation provided no benefit for preoperative planning compared with imaging on a 2D screen (Figure 3). In these cases, the rating of the facilitation of the preparation seemed to be most important for the overall evaluation. If this advantage was missing, the total benefit was clearly reduced (Figure 4). If there was no benefit for the representation of the pathology (e.g., intracardial) and the preparation (e.g., very superficial pathology), the holographic representation was overall inferior to the representation on a 2D screen.

Considering the responses of the surgeons to the patients in chronological order, no chronological dependency could be identified (Figure 3). The partly observed fluctuations in the graphics can be explained by patient-dependent weaknesses of the holographic representation. This rules out the possibility that the benefit of the method was seriously influenced by its first-time use as a new preoperative planning tool or by a habituation effect.

### CR-Holograms Led in Cost/Benefit Analysis

Looking at the costs and benefits of the evaluated methods, two different aspects must be considered: the cost (e.g., time expenditure)/benefit (e.g., reduction surgery time) balance for the surgeon in preoperative usage and the cost/benefit balance from a financial perspective of synthesizing the different types of 3D representations.

For the surgeon, CR-holograms as well as 3D-printed models provided a clear benefit in comparison with monitor-imaging (Table 2). The time required for both 3D imaging modalities was rated better than the time needed for 2D imaging, whereas CR-holograms were superior to 3D printing. The actual planning time was not measured due to the retrospective study design, but the surgeons' assessments revealed a clear cost-benefit advantage when 3D spatial representation was used for preoperative planning. Regarding their educational potential, both 3D-imaging methods were rated better than imaging on a 2D screen and thus equally useful. This finding was consistent with results from previous studies regarding the valuable educational potential of 3D printing (13, 14, 25, 26).

Nevertheless, the holographic representation was superior, considering the time expense. In the preparation process the cost/benefit of 3D printing is clearly inferior to CR-holograms. While the acquisition costs are similar for the 3D printer and the HoloLens®, the processing costs are much higher for 3D printing. Each print adds personnel and material costs. Furthermore, the intraoperative preparation time for a 3D print is much longer, which is why short-term production—for example, from an emergency CT for subsequent surgery—is not possible. In contrast, a CR-hologram can be prepared in little time.

### CR-Holograms Shorten the Intraoperative Preparation Time

To assess whether the subjectively identified benefit influenced the clinical outcome, we performed an objective evaluation of the spatial 3D-presentation methods by comparing the intraoperative preparation times between retrospectively matched pairs of patients. We found that the group that received preoperative planning based on 3D-printed models and CR-holograms showed significant reductions in the average intraoperative preparation time compared with the control group (cases: 58.5 ± 22.6 min vs. matched controls: 72.8 ± 43 min). Both spatial imaging techniques thus proved to be superior to standard imaging on 2D screen. We analyzed the intraoperative preparation time because our 3D-imaging methods are especially suitable for the representation of outer cardiovascular structures. Furthermore, as illustrated in Figure 4, the usefulness of the preoperative representation in reducing the intraoperative preparation time was decisive for the total benefit. We assumed that the 3D tissue imaging allows faster recognition of the outer structures, which makes a more precise and rapid preparation of the operative field possible. An assessment of the overall operative time would be influenced by a wide range of additional parameters, making the comparison much more difficult.

In nine patients the 3D imaging provided, according to the surgeon's opinion, no benefit to preparation time, independent of the reason for surgery. When examining the intraoperative preparation times of these patients, we identified no benefit relative to the matched patients (*P* = 0.59). Therefore, the subjective assessment by the surgeons corresponded to our objective findings regarding the preparation time. On the other hand, a significant reduction in the intraoperative preparation time could be measured in the “as advantageous” rated patients (*P* = 0.05). Though only a small patient cohort was analyzed, a clear difference was identified with slight significance. A few studies have suggested that using spatial 3D imaging preoperatively can improve surgical outcomes and reduce operative times in patients with CHD (15, 21, 27). However, a systematically measured significant shortening of the operative time has not before been determined. This is probably due to the fact that the overall operative time or the aortic cross-clamp time were analyzed, and no test of correlation with the surgeon's assessment was performed. For the first time, a significant positive influence of 3D spatial imaging in preoperative planning on the operative time in patients with CHD could be proven. Shortening of the overall operative time has already been shown to greatly influence postoperative outcome in cardiovascular surgery (28, 29). Nevertheless, the intraoperative preparation time is only one factor; many other factors, such as cross-clamp time and preoperative complications play an important role influencing overall operation time and outcome. To demonstrate this correlation, a prospective study with larger patient cohorts (e.g., multicenter study) is needed. This has the potential to improve the total outcome (e.g., by reducing postoperative complications and improving long-term survival). Since CR-holograms surpassed 3D printing in all analyzed subgroups, we assume that the benefit can be attributed mainly to the CR-holograms. It can therefore be concluded that detailed preoperative planning has a significant influence on the operation procedure, depending on the realism of the representation.

### Limitations

Due to the study design, the surgeon assessed the imaging material of the patient more intensively. A bias regarding the reduction of the intraoperative preparation time through this cannot be excluded. Additionally, the surgeon could not be blinded to the used imaging technique. Furthermore, since the patient pairs were matched retrospectively, it could not be granted that both patients were respectively treated by the same surgeon. A small patient cohort was used for analyses. The intraoperative benefit identified here was significant but should be validated in a larger study.

### Summary

In conclusion, this study demonstrated that spatial imaging provides a clear benefit in preoperative planning of pediatric heart surgery compared with the previously used representation on a 2D screen. The combination of an extremely photo-realistic surface representation by cinematic rendering and the presentation of the cardiovascular structures in 3D space improves the 3D perception enormously. This provides a better subjective assessment as well as a measurable shortening of the intraoperative preparation time. The cinematically rendered holographic presentation using mixed-reality glasses surpassed the previously used spatial 3D presentation method (3D printing) in all analyzed aspects. Therefore, it is reasonable to assume that the future of preoperative imaging lies in 3D-spatial representations, and particularly in CR-holograms.

### Prospects

There are clear limits to the representation of intracardial and intravascular structures since the current version of the HoloLens® only allows viewing of the outer surface without interactive cutting through the patient's heart. In the next version of the HoloLens®, an interactive cutting through the hologram will be possible. A further potential expansion of the presentation of CR-holograms would be a multi-user system that would enable joint discussion in 3D space. Furthermore, the visualization of hemodynamic information will likely be possible in future versions. The presentation of functional examinations (e.g., heart beating, 4D phase contrast, 4D speckle tracking) in 3D space is conceivable and would facilitate locating anatomical structures.

## Data Availability Statement

The raw data supporting the conclusions of this article will be made available by the authors, without undue reservation.

## Ethics Statement

Written informed consent was obtained from the individual(s) for the publication of any potentially identifiable images or data included in this article.

## Author Contributions

PG, RC, SD, and MA contributed to conception and design of the study. PG organized the database and performed together with MA the statistical analysis. PG, MA, RC, and AP were involved in the implementation of the study. SD and MA were responsible for the supervision of the study. PG and MA wrote the first draft of the manuscript. MS and SD reviewed and edited all sections of the manuscript. MU and OR provided cross-sectional imaging resources for the study. All authors read and approved the submitted manuscript version.

## Conflict of Interest

The authors declare that the research was conducted in the absence of any commercial or financial relationships that could be construed as a potential conflict of interest.
